# Enhancing interprofessional education: The role of direct communication in shaping student perceptions

**DOI:** 10.1016/j.jtumed.2025.02.015

**Published:** 2025-03-13

**Authors:** Ilham Khairi Siregar, Sefni Rama Putri, Palasara Brahmani Laras, Mohamad Awal Lakadjo

**Affiliations:** a Department of Guidance and Counseling, Universitas Negeri Gorontalo, Gorontalo, Indonesia; b Department of Psychology, Universitas Muhammadiyah Gorontalo, Gorontalo, Indonesia; c Department of Guidance and Counseling, Universitas Mercu Buana Yogyakarta, Daerah Istimewa Yogyakarta, Indonesia

**Keywords:** Healthcare collaboration, Interpersonal communication, Interprofessional education, Student perceptions, Teamwork in healthcare

Dear Editor,

We would like to highlight key findings from a recent study examining the impact of interprofessional education (IPE) on students' perceptions of healthcare roles. The research underscores the importance of IPE in fostering a more comprehensive understanding of professional responsibilities in the healthcare system. The study revealed that students who participated in a semester-long IPE program became more aware of the contributions of other healthcare professions, moving away from compartmentalized perceptions of professional roles.^1^

One of the most significant findings was the role of interpersonal communication in shaping student perceptions. Those who engaged in face-to-face interactions with peers from different disciplines reported more positive changes in their understanding of interprofessional collaboration. Conversely, students who relied solely on email communication demonstrated a higher tendency toward negative perceptions, often citing misunderstandings or perceived professional dominance.^2^ These results suggest that direct interpersonal engagement is crucial for fostering mutual respect and teamwork in healthcare education.

The implications of these findings for health professions education are profound. To optimize the benefits of IPE,^3^ institutions should prioritize structured opportunities for direct interaction, such as collaborative case discussions, simulation exercises, and joint clinical experiences.^4^ Furthermore, interprofessional learning should emphasize collaborative decision-making, ensuring that students develop the skills necessary for integrated, patient-centered care. Without intentional efforts to facilitate meaningful engagement, IPE initiatives may fail to produce the desired attitudinal and behavioral shifts needed for effective healthcare teamwork.

In conclusion, this study reinforces the value of well-designed interprofessional education programs that encourage direct communication and collaborative practice. As healthcare becomes increasingly interdisciplinary, fostering strong interprofessional relationships during training will be essential in preparing future professionals for the complexities of modern patient care.^5^ We encourage further exploration into innovative IPE strategies that enhance communication and teamwork among healthcare students.

## Source of funding

This research did not receive any funding from any organization, grant, or sponsorship.

## Conflict of interest

None.
